# Young donor hematopoietic stem cells revitalize aged or damaged bone marrow niche by transdifferentiating into functional niche cells

**DOI:** 10.1111/acel.13889

**Published:** 2023-05-24

**Authors:** Na Yuan, Wen Wei, Li Ji, Jiawei Qian, Zhicong Jin, Hong Liu, Li Xu, Lei Li, Chen Zhao, Xueqin Gao, Yulong He, Mingyuan Wang, Longhai Tang, Yixuan Fang, Jianrong Wang

**Affiliations:** ^1^ Research Center for Blood Engineering and Manufacturing Cyrus Tang Medical Institute, Suzhou Medical College of Soochow University Suzhou China; ^2^ State Key Laboratory of Radiation Medicine and Protection National Research Center for Hematological Diseases, Collaborative Innovation Center of Hematology, Soochow University Suzhou China; ^3^ The Department of Orthopedics The Affiliated Ninth Suzhou Hospital of Soochow University Suzhou China; ^4^ Institute of Blood and Marrow Transplantation, Jiangsu Institute of Hematology The First Affiliated Hospital of Soochow University Suzhou China; ^5^ Suzhou Blood Center Suzhou China

**Keywords:** aging, hematopoiesis, hematopoietic stem cells, stem cell niche, transdifferentiation

## Abstract

The bone marrow niche maintains hematopoietic stem cell (HSC) homeostasis and declines in function in the physiologically aging population and in patients with hematological malignancies. A fundamental question is now whether and how HSCs are able to renew or repair their niche. Here, we show that disabling HSCs based on disrupting autophagy accelerated niche aging in mice, whereas transplantation of young, but not aged or impaired, donor HSCs normalized niche cell populations and restored niche factors in host mice carrying an artificially harassed niche and in physiologically aged host mice, as well as in leukemia patients. Mechanistically, HSCs, identified using a donor lineage fluorescence‐tracing system, transdifferentiate in an autophagy‐dependent manner into functional niche cells in the host that include mesenchymal stromal cells and endothelial cells, previously regarded as “nonhematopoietic” sources. Our findings thus identify young donor HSCs as a primary parental source of the niche, thereby suggesting a clinical solution to revitalizing aged or damaged bone marrow hematopoietic niche.

## Graphical abstract

The bone marrow niche secures hematopoietic stem cells (HSCs) for hematopoiesis. Yuan et al. reported that young donor HSCs are able to regenerate aged or damaged bone marrow hematopoietic niche via transdifferentiation.

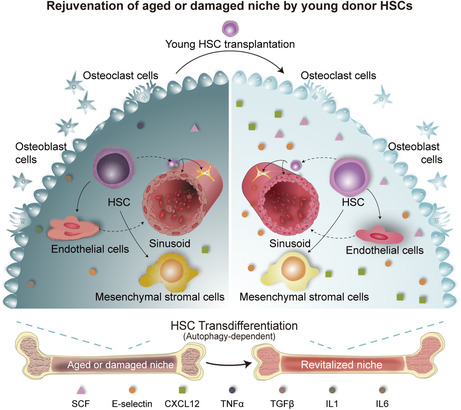

**Abbreviations**
ALL: acute lymphoblastic leukemia
AML: acute myeloid leukemia
Angpt1: Angiopoietin‐1
Atg7: autophagy‐related gene 7
BV/TV: trabecular relative bone volume
CXCL12: CXC‐chemokine ligand 12
CML: chronic myeloid leukemia
Ebf3: early B‐cell factor 3
EC: endothelial cell
HSC: hematopoietic stem cell
IR: irradiation
Micro‐CT: microcomputed tomography
MSC: mesenchymal stromal cell
OPN: osteopontin
PCA: principal component analysis
SCF: stem cell factor
Tb.Th: trabecular thickness
Tb.N: trabecular number
Tb.Sp: trabecular separation
Terf1: telomeric repeat binding factor 1
Terf2: telomeric repeat binding factor 2
Tert: telomerase reverse transcriptase

## 1 INTRODUCTION

The bone marrow HSC niche, initially proposed by Ray Schofield, is a local anatomical and functional microenvironment that consists of multiple cell populations and their secreted factors regulating hematopoietic stem cells (HSCs) for hematopoiesis (Méndez‐Ferrer et al., 2010; Morrison & Scadden, 2014; Scadden, 2014; Schofield, 1978; Yu & Scadden, 2016). Perturbation of the niche causes abnormal homing, quiescence, self‐renewal and differentiation of HSCs, and, ultimately, hematological disorders (Hooper et al., 2009; Oguro et al., 2013; Raaijmakers et al., 2010; Roberts et al., 2013; Zhang et al., 2012).

Early studies of the bone marrow niche have been primarily focused on the identification and function of its cellular and molecular components in governing HSCs. Mesenchymal stromal/stem cells (MSCs) and endothelial cells (ECs) are the two major niche members, which have been regarded as “nonhematopoietic” sources (Butler et al., 2010; Ding et al., 2012; Hooper et al., 2009; Kfoury & Scadden, 2015; Kiel et al., 2005; Kobayashi et al., 2010; Méndez‐Ferrer et al., 2010; Roberts et al., 2013; Winkler et al., 2012). Schwann cells and sympathetic neurons have also been suggested to be components of the bone marrow niche, supporting the function of HSCs (Arranz et al., 2014; Yamazaki et al., 2011). Additionally, osteoblastic cells and adipocytes are important for hematopoiesis despite their presence outside of the niche location (Calvi et al., 2003; Naveiras et al., 2009; Zhang et al., 2003; Zhou et al., 2017).

Niche cells modulate HSCs via the expression of niche factors, which include but are not limited to stem cell factor (SCF), stem cell location chemokine (CXCL12), and the glycoprotein (E‐selectin) (Ara et al., 2003; Dar et al., 2005; Ding & Morrison, 2013; Greenbaum et al., 2013; Nagasawa et al., 1996; Pinho et al., 2013; Sugiyama et al., 2006; Tzeng et al., 2011; Winkler et al., 2012; Zou et al., 1998). Although macrophages, megakaryocytes, and T cells are involved in mediating HSCs (Bruns et al., 2014; Crane et al., 2017; Winkler et al., 2010; Zhao et al., 2014), studies have reported that niche cells originate from nonhematopoietic sources. For example, the neuroepithelium and neural crest were suggested to supply MSCs and neural cells in the fetal bone marrow niche (Isern et al., 2014; Nagoshi et al., 2008; Takashima et al., 2007).

Hematopoiesis by HSCs involves their integration of intrinsic programs with extrinsic signals from the niche. Increased metabolism, decreased autophagy capacity, and altered epigenetic regulation are major intrinsic drivers of HSC aging (Chandel et al., 2016; Fang et al., 2020; Ho et al., 2017; López‐Otín et al., 2016; Verovskaya et al., 2019). Recent studies have overwhelmingly investigated the impact of the niche on HSCs and hematopoiesis. Bone marrow hematopoietic niche aging is characterized by degeneration of adrenergic nerves in bone marrow (Maryanovich et al., 2018), increased inflammatory signals (Verovskaya et al., 2019), and deregulated proliferation of stromal cells with reduced niche factors (Pinho & Frenette, 2019; Verovskaya et al., 2019). Niche aging promotes the transformation of HSCs and the development of malignant hematological disorders (Curto‐Garcia et al., 2020; Duarte et al., 2018; Gnani et al., 2019; Hurwitz et al., 2020; Shlush, 2018; Zhan & Kaushansky, 2020). Niche function can be improved by engineered MSCs with the transcription factors Klf7, Ostf1, Xbp1, Irf3, and Irf7 (Nakahara et al., 2019), and long‐term engraftment of primary bone marrow stromal cells repairs niche damage and improves HSC transplantation (Abbuehl et al., 2017). The young niche in the recipient mice is able to largely restore the transcriptional profile of aged donor HSCs but not their DNA methylation profiles. Therefore, restoration of the young niche is insufficient for rejuvenating HSC function (Kuribayashi et al., 2021).

However, studies on the active role of HSCs in reciprocally shaping the stem cell niche are still lacking. In particular, the parental source and its underlying mechanisms by which bone marrow niche cells are regenerated or repaired in mammalian adults remain fundamentally unexplored. This study attempted to address these issues.

## 2 RESULTS

### 2.1 HSC dysfunction by autophagy disruption led to an aging‐like bone marrow niche lacking niche factors

**FIGURE 1:**
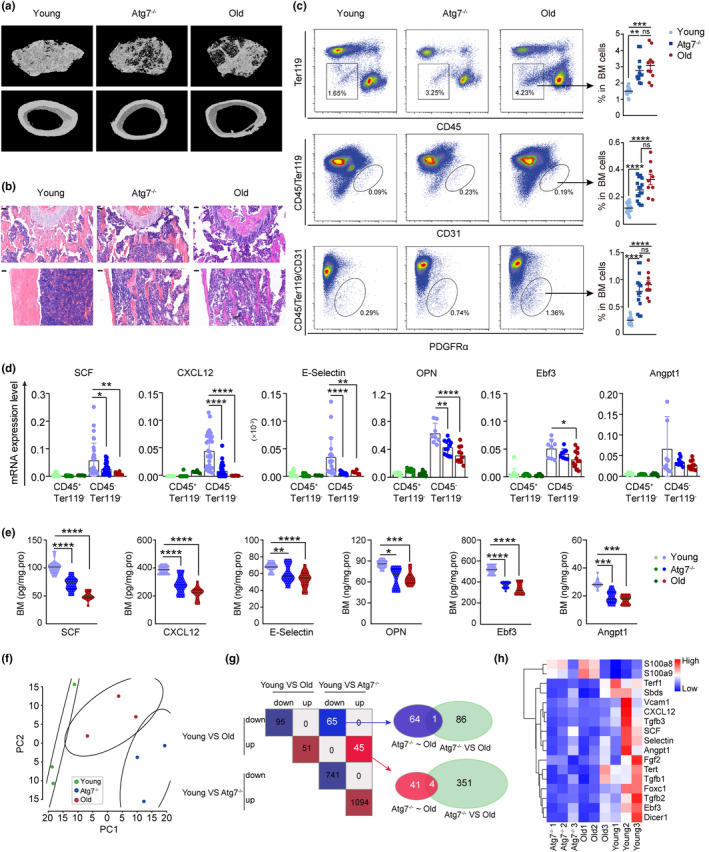
Deletion of the autophagy gene *Atg7* led to an abnormal bone marrow microenvironment and niche cell populations lacking niche factors, comparable to those in old mice. (a) Representative micro‐CT reconstructed three‐dimensional pictures of femur trabecular (top) and cortical bone (bottom). Femora were collected from 10‐week‐old mice, 72‐week‐old mice and 10‐week‐old *Atg7* ^−/−^ mice. *n* = 3, from three independent experiments. (b) H&E staining of femur paraffin sections. Femurs were collected from young mice (10‐week‐old), *Atg7* ^−/−^ mice (10‐week‐old), and old mice (72‐week‐old) for paraffin sectioning and immunohistochemistry. Bar = 50 μm. *n* = 3, from three independent experiments. (c) Representative flow cytometry plots (left) with quantification of CD45^−^Ter119^−^cells, CD45^−^Ter119^−^CD31^+^ cells, and CD45^−^Ter119^−^CD31^−^PDGFRα^+^ cells in the bone marrow of young, *Atg7* ^−/−^, and old mice (right). (d) The transcriptional levels of niche factors in hematopoietic cells (CD45^+^Ter119^−^) and the bone marrow stroma cells (CD45^−^Ter119^−^) were detected by quantitative real‐time PCR. (e) Protein levels of niche factors in bone marrow were quantified by ELISA. (f) PCA summarizing the top 2000 most variable genes among the gene expression profiles of young, old and *Atg7* ^−/−^ mouse MSCs. (g) Left panel, intersection of differentially expressed genes in two comparison schemes (young vs. old and young vs. *Atg7* ^*−/−*^). Right panel, Venn diagram between the intersecting gene sets from the left panel and differentially expressed genes from old versus *Atg7* ^−/−^ mice. (h) Heatmap depicting the expression of niche factors in MSC transcriptomic profiling. Expression levels are row‐normalized. ^ns^ *p* > 0.05; *p < 0.05; **p < 0.01; ***p < 0.001; ****p < 0.0001; unpaired two‐tailed *t* test. All error bars indicate SD.

To explore the role of HSCs in the bone marrow niche, we employed a conditional mouse model (*Atg7* ^f/f^;Vav‐iCre) in which HSC function is impaired and features a speedy aging‐like phenotype in hematopoiesis due to the selective deletion of the autophagy‐essential gene *Atg7* (Cao et al., 2015; Fang et al., 2020; Mortensen et al., 2011). Dysfunction of HSCs in 10‐week‐old mice led to an abnormal bone marrow architecture, manifested as an obvious loss of trabecular structures and apparent cortical thinning in the *Atg7* ^−/−^ mice observed in microcomputed tomography scanning images (Figure 1a); note that the architecture of the 10‐week‐old *Atg7* ^−/−^ mice appeared similar to the bone marrow of 72‐week‐old mice. Hematoxylin–eosin staining revealed a severe loss of trabecular and cortical bone in the femora and tibiae of *Atg7* ^−/−^ mice, similar to 72‐week‐old mice (Figure 1b). Flow cytometry of bone marrow niche cells indicated an aberrant increase in the proportion of bone marrow stromal cells, endothelial cells (ECs), and mesenchymal stromal cells (MSCs) in the bone marrow of *Atg7* ^−/−^ mice (Figure 1c). Examination of 10‐week‐old *Atg7* ^−/−^ mice showed significantly reduced transcription of a group of niche factors (SCF, CXCL12, E‐selectin, OPN, Ebf3, and Angpt1) in bone marrow stromal cells (CD45^−^Ter119^−^cells) but not hematopoietic cells (CD45^+^Ter119^−^ cells) (Figure 1d). ELISA results from the *Atg7* ^−/−^ mice also showed significantly reduced protein levels of the major niche factors (Figure 1e). All of these phenotypes are often seen in aged bone marrow niche with declined hematopoiesis (Verovskaya et al., 2019).

To further examine the impact of HSCs on the nature of bone marrow niche cells, we performed transcriptomic profiling of MSCs, which revealed the top 2000 most variable genes in expression levels of young, old and *Atg7* ^−/−^ mice (Figure 1f). Upregulation or downregulation of differentially expressed genes in young versus old mice was similar to that in young versus *Atg7* ^−/−^ mice (Figure 1g). Unlike young mice, where bone marrow MSCs express a long list of high levels of niche factors, in old and *Atg7* ^−/−^ mice, their MSCs express a significantly reduced level of niche factors (Figure 1h). These data suggest that the transcriptional profiles of niche cells of HSC‐dysfunctional *Atg7* ^−/−^ mice resemble those of physiologically aged mice. Therefore, the above results indicate that HSCs are essential in counteracting niche aging and maintaining niche function and that *Atg7* ^−/−^ mice bear an aged or declined bone marrow hematopoietic niche.

### 2.2 Transplantation of young but not aged HSCs restored the genetically or physically damaged bone marrow niche and rejuvenated the aged bone marrow niche in mice

**FIGURE 2:**
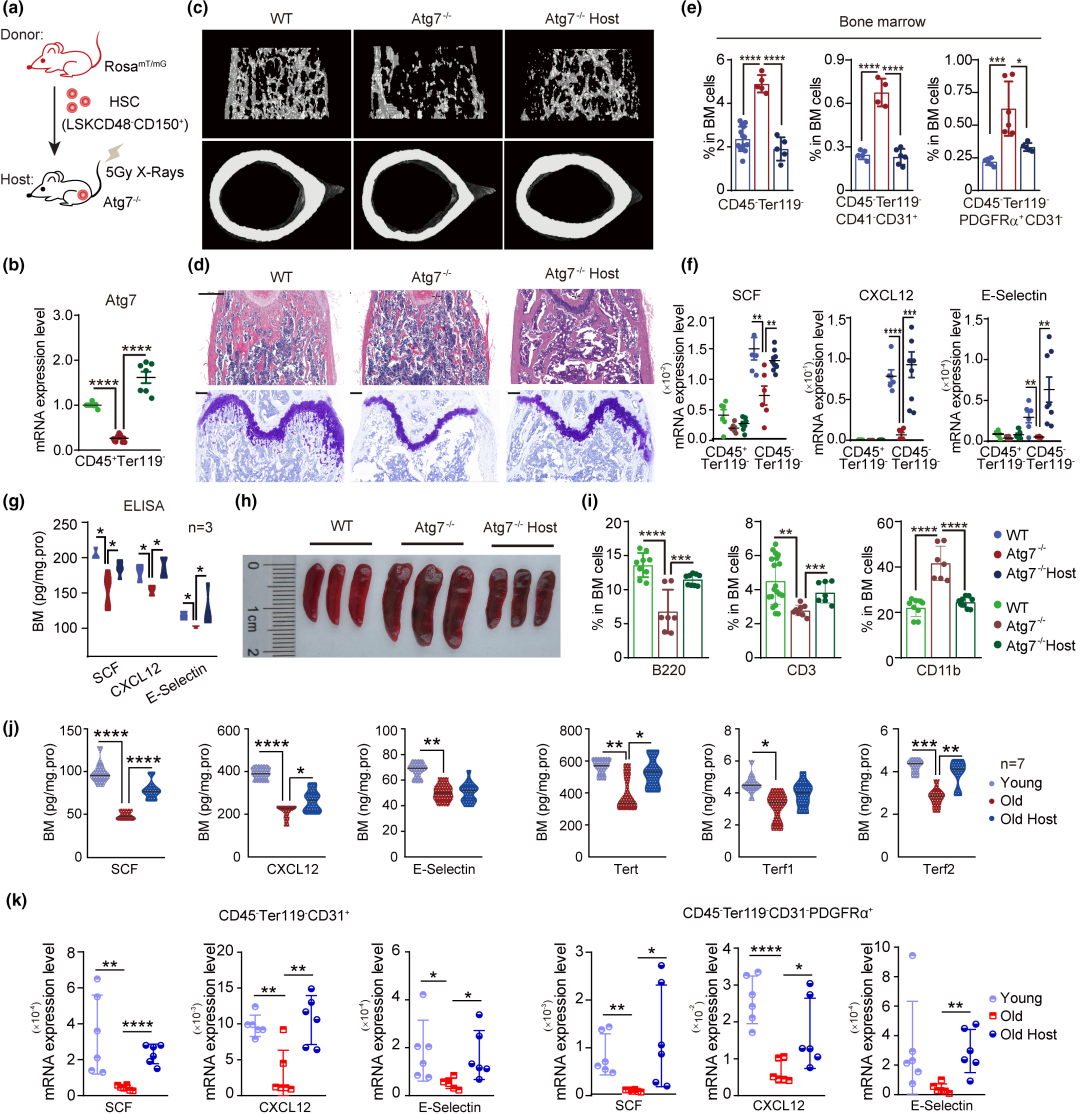
Young HSCs rebuilt the bone marrow “nonhematopoietic” niche and reconstituted hematopoietic cells in the host of the endogenous bone marrow niche predisrupted with irradiation or restored the levels of niche factors and antiaging telomerases in the bone marrow stromal cells of the old host. (a) Schematic procedure of the transplantation. *Atg7* ^−/−^ mice irradiated with 5 Gy were transplanted with 2000 young donor HSCs from *Rosa* ^*mT/mG*^ mice and 200,000 whole bone marrow cells from CD45.1 mice. The host bone marrow cells were analyzed at 16 weeks posttransplantation. (b) Detection of young donor *Atg7* expression in CD45^+^Ter119^−^ cells from WT, *Atg7* ^−/−^, and *Atg7* ^−/−^ host mice. *Atg7* transcripts in host CD45^+^Ter119^−^ cells after HSC transplantation were detected by quantitative real‐time PCR. (c) Representative micro‐CT reconstructed three‐dimensional pictures of femur trabecular (top) and cortical bones (down). Femora were collected from 10‐week‐old WT, *Atg7* ^−/−^ and *Atg7* ^−/−^ host mice. *n* = 3, from three independent experiments. (d) Upper, H&E staining of femur paraffin sections (scale bar, 500 μm). Bottom, toluidine blue staining (scale bar, 200 μm). Femurs were collected from 10‐week‐old WT, *Atg7* ^−/−^ and *Atg7* ^−/−^ host mice for paraffin sectioning and immunohistochemistry. *n* = 3, from three independent experiments. (e) Percentages of CD45^−^Ter119^−^ cells, CD45^−^Ter119^−^CD31^+^ cells, and CD45^−^Ter119^−^CD31^−^PDGFRα^+^ cells in the bone marrow of WT, *Atg7* ^−/−^ and *Atg7* ^−/−^ host mice were analyzed by flow cytometry. (f) Restored expression levels of niche factors from bone marrow cells in WT, *Atg7* ^−/−^, and *Atg7* ^−/−^ host mice. The transcription of niche factors in hematopoietic (CD45^+^Ter119^−^) cells and stromal cells (CD45^−^Ter119^−^) was detected by quantitative real‐time PCR. (g) Protein levels of niche factors in the bone marrow were quantified by ELISA (*n* ≥ 3). (h) Representative spleen size in the indicated WT, *Atg7* ^−/−^, and *Atg7* ^−/−^ host mice. (i) The proportions of B220^+^ cells, CD3^+^ cells, and CD11b^+^ cells in the bone marrow from WT, *Atg7* ^−/−^, and *Atg7* ^−/−^ host mice were measured by flow cytometry. (j) Protein levels of three niche factors (SCF, CXCL12, and E‐selectin) and three telomerases (Tert, Terf1, and Terf2) in the bone marrow of 72‐week‐old mice were quantified by ELISA. (k) Restored expression levels of niche factors from bone marrow cells in young, old, and old host mice. The transcription of niche factors in EC cells (CD45^−^Ter119^−^CD31^+^) and MSC cells (CD45^−^Ter119^−^CD31^−^PDGFRα^+^) was detected by quantitative real‐time PCR. ^ns^ *p* > 0.05; *p < 0.05; **p < 0.01; ***p < 0.001; ****p < 0.0001; unpaired two‐tailed *t* test. All error bars indicate SD.

To examine whether young donor HSCs are able to regenerate a functional hematopoietic niche in the host bone marrow whose niche is genetically predisposed, we transplanted 2000 HSCs from young *Rosa* ^mT/mG^ mice (8 weeks old) with 0.2 million bone marrow cells from wild‐type nontransgenic mice to host *Atg7* ^−/−^ mice (Figure 2a). Restoration of the *Atg7* expression level in the host bone marrow CD45^+^Ter119^−^ cells of *Atg7* ^−/−^ mice (Figure 2b) suggests successful homing of normal donor HSCs to the predisrupted bone marrow niche. Sixteen weeks after HSC transplantation, the impaired bone marrow structure in the *Atg7* ^−/−^ mice was reversed close to the wild‐type levels (Figure 2c,d). In particular, the cellularity of stromal cells and two major niche cell members, ECs and MSCs, abnormally proliferated in *Atg7* ^−/−^ mice and were well normalized to the levels of the wild‐type niche after HSC transplantation (Figure 2e). Furthermore, the expression of three major niche factors (SCF, CXCL12 and E‐selectin) in bone marrow stromal cells, which were previously reduced in *Atg7* ^−/−^ mice,  was also rescued in the *Atg7* ^−/−^ host at both the transcriptional and translational levels (Figure 2f,g), strongly indicating functional restoration of the bone marrow “nonhematopoietic” niche in the host from young donor HSCs. In accordance with the above results, extramedullary hematopoiesis in *Atg7* ^−/−^ mice was reversed to normal bone marrow hematopoiesis as the spleen size was normalized (Figure 2h), and multilineage differentiation was restored since the proportion of myeloid and lymphoid cells returned to normal (Figure 2i).

To support the observation of the HSC capacity to restore the “nonhematopoietic” niche that was genetically disrupted by deletion of *Atg7* in the hematopoietic system, we also examined the capacity of young donor HSCs to restore the niche destroyed by irradiation (Figure S1a). Irradiation destroyed the whole hematopoietic system and bone marrow microenvironment, thus intensively reducing the proportion of stromal and hematopoietic cells in the mouse. However, when young HSCs were transplanted, the proportion of niche cells was effectively restored in the irradiated host (Figure S1b). Microcomputed tomography scanning of the irradiated mice exhibited a severe decrease in trabecular relative bone volume (BV/TV), trabecular number, cortical bone density and cancellous bone density, as well as a significant elevation of trabeculae separation, and hematoxylin–eosin staining of femurs also showed abnormal bone marrow morphology; young donor HSCs reversed the bone destruction, as evidenced by obviously improved trabeculae separation and cancellous bone density after young HSCs were transplanted into the irradiated mice for 16 weeks (Figure S1c,d).

To examine whether young donor HSCs are able to regenerate functional niche in physiologically aged niche in host mice, HSCs from 8‐week‐old mice were transplanted into 72‐week‐old mice. As expected, three major niche factors (SCF, CXCL12, and E‐selectin) and three telomerases (Tert, Terf1, and Terf2), which are antiaging markers in bone marrow stromal cells that were previously reduced in old mice, were rescued in the 72‐week‐old host at the protein level (Figure 2j). Furthermore, expression of the niche factors in sorted niche cell populations MSCs (CD45^−^Ter119^−^CD31^−^PDGFRα^+^) and ECs (CD45^−^Ter119^−^CD31^+^) was also rescued in the 72‐week‐old host at mRNA level (Figure 2k). Together, these data suggest rejuvenation of the bone marrow “nonhematopoietic” niche in the physiologically aged host by young donor HSCs.

**FIGURE 3:**
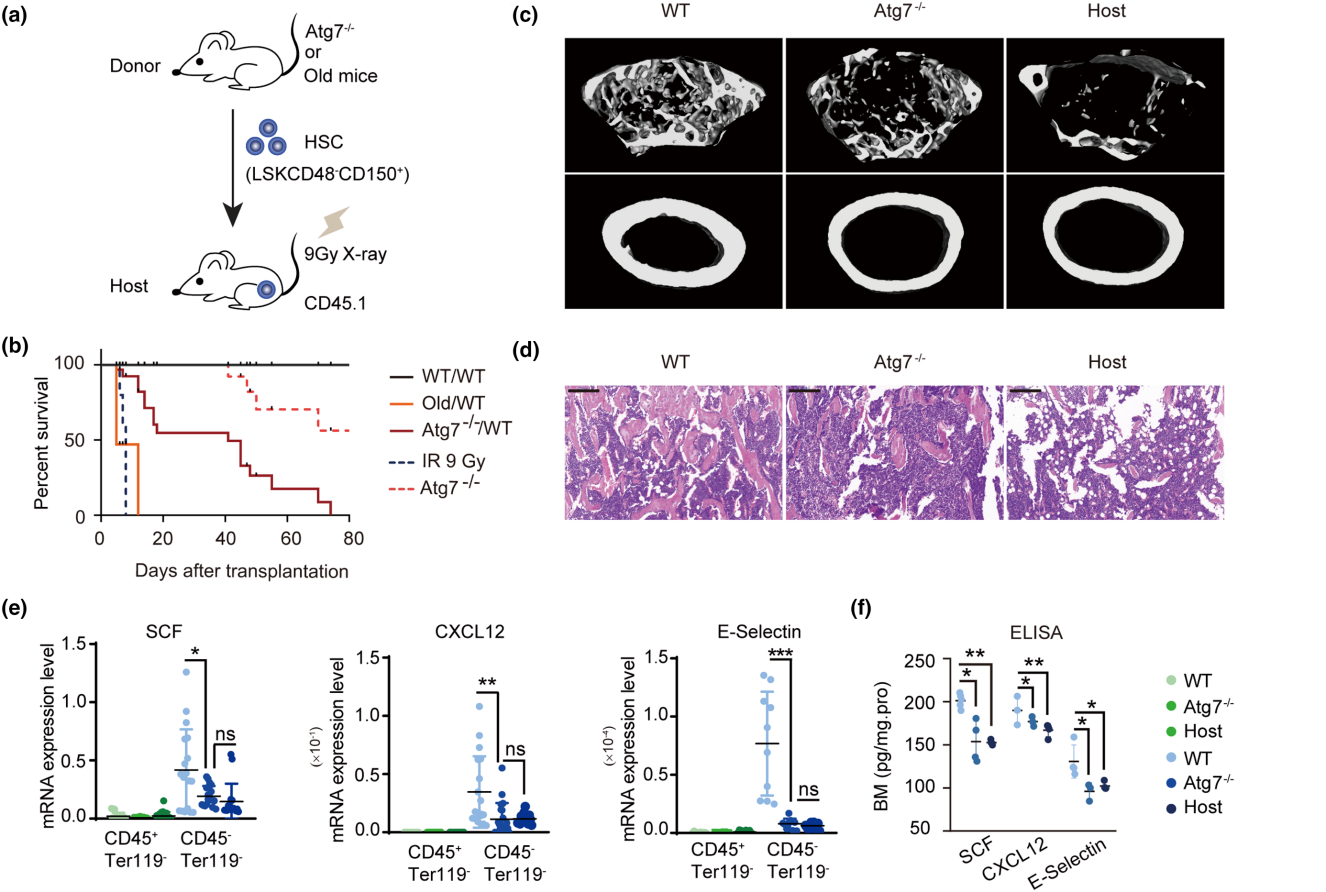
Aged HSCs cannot reconstruct the bone marrow niche previously disrupted by irradiation. (a) Schematic procedure of HSC transplantation. Nine Gy‐irradiated CD45.1 mice were transplanted with 2000 HSCs and 200,000 whole bone marrow cells from *Atg7* ^f/f^;Vav‐iCre mice. The host bone marrow cells were analyzed at 16 weeks posttransplantation. (b) The survival curves of WT/WT, Old/WT, *Atg7* ^*−/−*^/WT, *Atg7* ^*−/−*^, and irradiated mice. (c) Representative micro‐CT reconstructed three‐dimensional pictures of femur trabecular (top) and cortical bones (down). Femora were collected from 10‐week‐old WT, *Atg7* ^*−/−*^ and host mice. *n* = 3, from three independent experiments. (d) H&E staining of femur paraffin sections. Femurs were collected from 10‐week‐old WT, *Atg7* ^*−/−*^ and host mice for paraffin sectioning and H&E staining. Bar = 200 μm. (*n* = 3 mice from three independent experiments). (e,f) Expression analysis of niche factors from bone marrow cells in WT, *Atg7* ^*−/−*^, and *Atg7* ^*−/−*^ host mice. The transcription of niche factors for SCF, CXCL12, and E‐selectin in hematopoietic (CD45^+^Ter119^−^) cells and nonhematopoietic cells (CD45^−^Ter119^−^) was detected by quantitative real‐time PCR (left). Protein levels of SCF, E‐selectin and CXCL12 in bone marrow were quantified by ELISA (right). ^ns^ *p* > 0.05; *p < 0.05; **p < 0.01; ***p < 0.001; ****p < 0.0001; unpaired two‐tailed *t* test. All error bars indicate SD.

However, when we transplanted *Atg7* ^−/−^ HSCs, phenotypically aged HSCs with autophagy defects (Fang et al., 2020), into irradiated mice (Figure 3a), all of them died within 80 days after transplantation (Figure 3b). Similarly, when we transplanted old HSCs from 48‐month‐old mice into irradiated mice, all of them died within 20 days after transplantation (Figure 3b). The bone structure (Figure 3c,d) and the expression levels of the niche factors SCF, CXCL12 and E‐selectin were not improved until the mice died (Figure 3e,f). Therefore, young but not aged or dysfunctional donor HSCs are able to restore the “nonhematopoietic” niche, and restoration of the niche depends on the integrity of the autophagy machinery.

### 2.3 Host bone marrow nonhematopoietic niche cells carried markers from young donor HSCs

**FIGURE 4:**
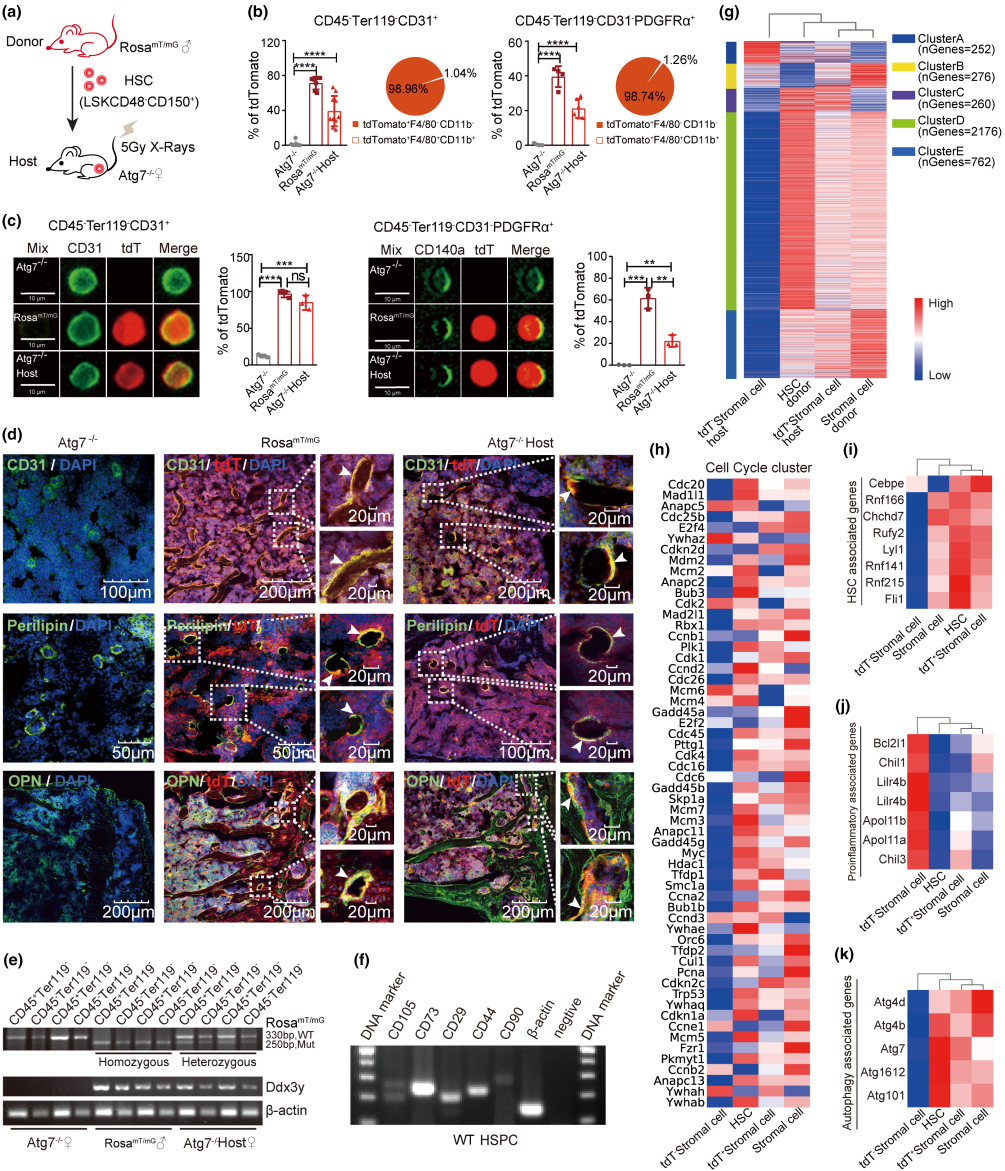
Host bone marrow “nonhematopoietic” niche cells predamaged by autophagy disruption carried the markers and transcriptomic imprinting of young donor HSCs. (a) Schematic protocol of the transplantation. *Atg7* ^−/−^ mice irradiated with 5 Gy were transplanted with 2000 HSCs from *Rosa* ^*mT/mG*^ mice and 200,000 whole bone marrow cells from *Atg7* ^−/−^ mice. The host bone marrow cells were analyzed at 16 weeks posttransplantation. (b) The percentages of bone marrow tdTomato^+^ cells in CD45^−^Ter119^−^CD31^+^ cells and CD45^−^Ter119^−^CD31^−^PDGFRα^+^ cells from the indicated mice were detected by flow cytometry (histogram). The percentages of cells with F4/80 and CD11b double‐positive signals in the indicated tdTomato^+^CD45^−^Ter119^−^CD31^+^ cells and tdTomato^+^CD45^−^Ter119^−^CD31^−^PDGFRα^+^ cells were detected by flow cytometry (pie chart). (c) Imaging flow analysis of CD45^−^Ter119^−^CD31^+^ cells and CD45^−^Ter119^−^CD31^−^PDGFRα^+^ cells from *Atg7* ^−/−^, *Rosa* ^*mT/mG*^, *Atg7* ^−/−^ host mice (left). CD45^−^Ter119^−^CD31^+^ cells (green dots), *Rosa* ^*mT/mG*^ fluorescence (tdT) (red dots), merge (yellow dots). Quantification of merged cells in the indicated mice 16 weeks after transplantation (right). (d) Representative confocal images of bones from *Atg7* ^−/−^, *Rosa* ^*mT/mG*^, and *Atg7* ^−/−^ host mice were stained for CD31^+^ vasculature endothelial cells, Perilipin^+^ adipocytes, OPN^+^ osteoblasts and 4′,6‐diamidino‐2‐phenylindole (DAPI). High magnifications (right) show the enlarged colocalization regions. Arrows mark the merged cells. (e) PCR analysis of the presence of the *Ddx3y* gene in CD45^−^Ter119^−^ cells and CD45^+^Ter119^−^ cells in *Atg7* ^−/−^ female, *Rosa* ^*mT/mG*^ male, and *Atg7* ^−/−^ host female mice. *Atg7* ^−/−^ female mice served as a negative control, and *Rosa* ^*mT/mG*^ male mice served as a positive control. (f) HSPCs sorted against Lin^−^Sca‐1^+^c‐Kit^+^ from the bone marrow of WT mice were prepared for subsequent RNA extraction and reverse transcription‐PCR assay. *n* = 3, from three independent experiments. (g–k) RNA sequencing with donor HSCs and host tdTomato^−^ stromal cells, host tdTomato^+^ stromal cells and total stromal cells of the donor bone marrow. All expression data in the heatmap are row normalized. Heatmap illustrating clusters of genes with high abundance in transcripts. Columns/samples were clustered using complete‐linkage clustering, and rows/genes were clustered using kmeans (See method for details) (g); heatmap illustrating transcription profile of genes related to cell cycle (h); heatmap illustrating the transcription profile of genes related to HSC signatures (i); heatmap illustrating the transcription profile of proinflammatory genes (j); heatmap illustrating the expression profile of genes related to autophagy (k). tdT: tdTomato; ^ns^ *p* > 0.05; *p < 0.05; **p < 0.01; ***p < 0.001; ****p < 0.0001; unpaired two‐tailed *t* test. All error bars indicate SD.

To study how young donor HSCs contribute to regeneration of the bone marrow niche, the “nonhematopoietic” cells, we used young donor HSCs of *Rosa* ^mT/mG^ mice to serve as a cell tracing system in a host since this animal has universal expression of tdTomato fluorescence in all tissues; hence, the daughter cells from young donor HSCs of *Rosa* ^mT/mG^ mice can be tracked via tdTomato fluorescence. We transplanted 2000 *Rosa* ^mT/mG^ HSCs with 0.2 million CD45.1 bone marrow cells into *Atg7* ^−/−^ mice or lethally irradiated mice via tail vein injection to examine the reconstitution capacity of HSCs (Figures 4a and S1a). Compared with the nontransplant control, we found that more than 40% of ECs and approximately 20% of MSCs had tdTomato fluorescence in host mice (Figure 4b). ImageStream measurement quantified over 90% colocalization between tdTomato fluorescence and CD31 (from CD45^−^Ter119^−^) and approximately 25% colocalization between tdTomato and PDGFRα (from CD45^−^Ter119^−^CD31^−^) (Figure 4c), suggesting that these niche cells may originate from donor HSCs. Furthermore, imaging of ECs (CD31) and MSC‐derived adipocytes (Perilipin) and osteoblasts (OPN) in the host bone marrow also revealed colocalization with tdTomato fluorescence in these cells, again supporting that HSCs are the source of these niche cells (Figure 4d).

Next, we used quantitative PCR to trace the male‐specific gene *Ddx3y* on the Y chromosome of the young donor HSCs of male mice in the “nonhematopoietic” niche cells of the recipient female mice. The CD45^−^Ter119^−^ stromal cells of the *Atg7* ^−/−^ female host acquired the *Ddx3y* gene, similar to the CD45^+^Ter119^−^ hematopoietic cells carrying *Ddx3y* from the donor HSCs (Figure 4e). Bone marrow CD45^−^Ter119^−^ stromal cells have long been regarded as “nonhematopoietic.” However, it appears here that HSCs are transdifferentiated to these niche cells. This notion is further supported by the fact that the *Rosa* ^mT/mG^ genotype in CD45^−^Ter119^−^ stromal cells was changed from homozygous (in the donor) to heterozygous (in the host) (Figure 4e). Apart from analysis of the generation of the niche predisrupted by *Atg7* deletion, we also observed that the host bone marrow niche cells, predamaged by irradiation, carried markers of young donor HSCs (Figure S2). Thus, young donor HSCs are transdifferentiated to the “nonhematopoietic” niche populations in the host whose bone marrow niche was previously damaged by autophagy disruption or irradiation.

MSCs express cell markers, such as CD105, CD73, CD90, and CD29 and are always positive for CD44 and negative for CD45 (Kfoury & Scadden, 2015; Su et al., 2014). We examined these MSC‐specific markers in the HSCs of nontransplanted wild‐type mice and found that HSC‐enriched hematopoietic cells (HSPCs) also express these markers (Figure 4f), and a previous study found that HSCs express Ang1, an EC marker, to regulate their niche (Zhou et al., 2015). These data together suggest that under physiological conditions, bone marrow MSCs and ECs may also originate from bone marrow HSCs.

### 2.4 Host bone marrow nonhematopoietic niche cells carried transcriptomic imprinting from young donor HSCs

To extend the identification of the niche cells from tracing single markers to a wider expression landscape, we performed transcriptomic profiling, which clearly indicates that while substantial contrast exists between donor HSCs and tdTomato^−^ bone marrow stromal cells that are nondonor source, the transcriptomic pattern of tdTomato^+^ stromal cells in the host that was transplanted with HSCs apparently lies between the donor HSCs and tdTomato^−^ stromal cell population. With particular comparisons in five clusters for transcription, HSCs are characterized by high expression in cluster D, which includes 2176 transcripts, and these transcripts can partially be seen in the stromal cells (tdTomato^+^ stromal cells) of the host after HSC transplantation but not in the tdTomato^−^ stromal cells that do not carry fluorescence from donor HSCs; note that the stroma cells in the last lane were from wild‐type young mice revealing active expression profiles (Figure 4g).

In contrast, the tdTomato^−^ bone marrow stromal cells displayed approximately 80% silenced genes in a total of 3726 transcripts enriched in the transcriptomic profiling, suggesting that the majority of the tdTomato^−^ niche cells are silenced. tdTomato^−^ niche cells expressed only 252 transcripts in cluster A, but these transcripts were lower in HSCs and HSC‐regenerated niche cells. Likewise, the tdTomato^−^ niche cells did not express transcripts in cluster E (762 transcripts), but HSCs and HSC‐regenerated niche cells were expressed at relatively high levels. In particular, the gene expression profile in HSCs showed similar patterns in regenerated niche cells (Figure 4g). The tdTomato^−^ niche cells that were not derived from the donor HSCs displayed severe inactivation of cell cycle regulators compared to donor HSCs and HSC‐regenerated niche cells (Figure 4h), again suggesting that the niche cells of the non‐HSC source, that is, tdTomato^−^ cells, are aged niche populations driven by extensive silencing.

A group of genes active in HSCs was also expressed in HSC‐regenerated niche cells (Figure 4i) but not in tdTomato^−^ cells, which are not of HSC origin. A group of proinflammatory genes and proapoptotic genes highly expressed in the tdTomato^−^ stromal cells were very lowly expressed in donor HSCs and fairly lowly expressed in the stromal cells excluding tdTomato^−^ stromal cells (Figure 4j). High autophagy activity is associated with juvenescence (Ho et al., 2017), and autophagy is significantly downregulated in aged HSCs (Fang et al., 2020) and aged bone marrow (Yuan et al., 2020). Consistent with this finding, aged tdTomato^−^ stromal cells displayed low expression of autophagy‐associated genes, but stromal cells, excluding tdTomato^−^ stromal cells, expressed high levels of autophagy‐related genes as donor HSCs (Figure 4k), suggesting that host niche cells were rejuvenated after HSC transplantation.

Together, based on the identification of donor single markers and transcriptomic patterns in the host niches, the host nonhematopoietic niche cells MSCs and ECs carry the HSC donor markers, and the regenerated niche displays a distinct expression pattern overlapping with donor HSCs but different from the host own aged niche cells. Therefore, the above results support the notion that “nonhematopoietic” niche cells are derived from donor HSCs via a transdifferentiating trajectory and become rejuvenated.

### 2.5 HSC‐derived “nonhematopoietic” niche cells in the host can be excluded from macrophage‐mediated phagocytosis or cell fusion between donor HSCs and niche cells

To examine whether the specific markers and transcriptomic patterns of donor HSC origin that appeared in the regenerated or repaired niche cells in the host bone marrow were possibly caused by cellular cascades other than transdifferentiation, we analyzed the flow cytometric data on the frequency of donor fluorescence in the host mice. In host *Atg7* ^−/−^ mice or lethally irradiated mice posttransplantation of donor *Rosa* ^mT/mG^ HSCs and supporting bone marrow cells, 98.96% of tdTomato^+^ CD45^−^Ter119^−^CD31^+^ (EC) cells are F4/80^−^CD11b^−^, and 98.74% of tdTomato^+^CD45^−^Ter119^−^CD31^−^PDGFRα^+^ (MSC) cells are F4/80^−^CD11b^−^, excluding the possibility of phagorytosis of donor HSCs by macrophages (F4/80^+^CD11b^+^) to generate fluorescent niche cells (Figure 4b).

**FIGURE 5:**
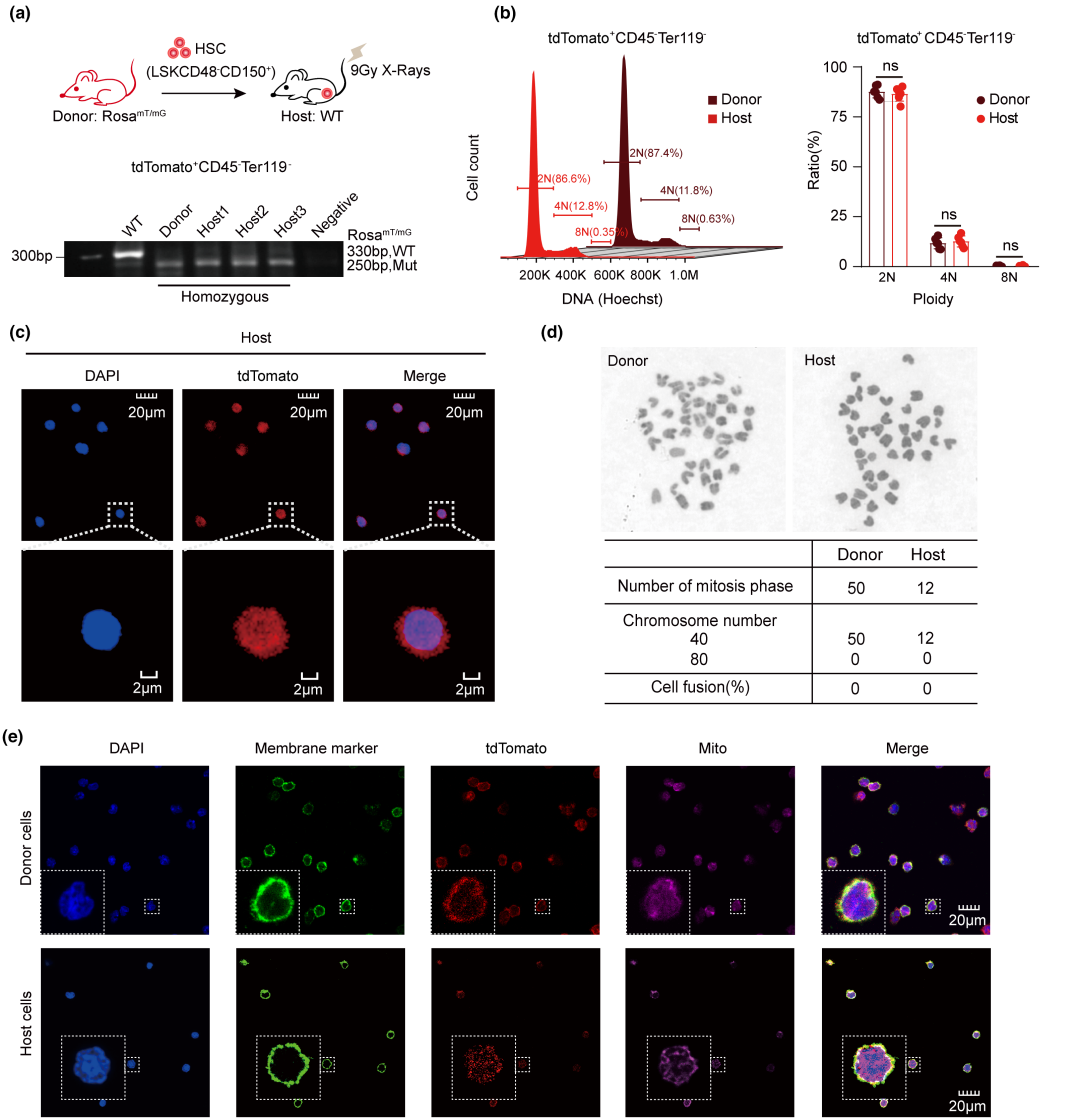
Aged or damaged bone marrow niche can be restored by donor HSCs, not via phagocytosis or cell fusion. (a) Genotyping of *Rosa* ^mT/mG^ and host mice. PCR analysis of *Rosa* ^mT/mG^ genotype from tdTomato^+^CD45^−^Ter119^−^ cells in host mice. *Rosa* ^mT/mG^ mice served as a positive control. Wild‐type mice served as a negative control. *n* = 3, from three independent experiments. (b) Detection of DNA ploidy. tdTomato^+^ CD45^−^Ter119^−^ cells in the bone marrow from *Rosa* ^mT/mG^ and host mice were stained with Hoechst and subjected to FACS analysis. Left, representative flow cytometry plots; right, DNA ploidy statistics. (c) Confocal detection of coenocytes. Representative confocal images of tdTomato^+^CD45^−^Ter119^−^ cells from host mice stained for 6‐diamidino‐2‐phenylindole (DAPI). (d) Karyotype FISH of *Rosa* ^mT/mG^ and host mice. Chromosome count from donor *Rosa* ^mT/mG^ and host mice. The above image shows the number and morphology of chromosomes at metaphase, and the following table shows the statistics of chromosome number and the percentage of cell fusion in *Rosa* ^mT/mG^ and host mice. (e) Representative confocal images of donor and host cells stained with anti‐sodium potassium ATPase antibody (membrane marker), MitoTracker (mitochondrial marker), and 4,6‐diamidino‐2‐phenylindole (DAPI). ^ns^ *p* > 0.05; *p < 0.05; **p < 0.01; ***p < 0.001; ****p < 0.0001; unpaired two‐tailed *t* test. All error bars indicate SD.

To test whether the regenerated “nonhematopoietic” niche cells carrying tdTomato fluorescence were derived from cell‐cell fusion between the donor HSCs and nonhematopoietic niche cells of the host source, we transplanted HSCs from *Rosa* ^mT/mG^ mice to irradiated wild‐type mice. Bone marrow stromal cells (tdTomato^+^CD45^−^Ter119^−^) from the host mice showed a homozygous *Rosa* ^mT/mG^ genotype by PCR amplification, consistent with that of the donor *Rosa* ^mT/mG^ mice, and no hybrid genotype between donor HSCs and host nonhematopoietic cells was found (Figure 5a). tdTomato^+^CD45^−^Ter119^−^ cells were transdifferentiated from donor mice showing *Rosa* ^*mT/mG*^ homozygous genotyping. Furthermore, tdTomato^+^CD45^−^Ter119^−^ cells from host mice were stained with Hoechst and analyzed for chromosome ploidy by FACS, and no polyploidy was found in these HSC‐derived cells (Figure 5b), which strongly indicates that the niche cells of host mice originated from donor HSCs and not from cell–cell fusion. Next, DAPI staining was performed on the nuclei, and fluorescence microscopy showed that there were no multinucleated cells (Figure 5c), again supporting the lack of cell‐cell fusion. To further examine whether the number of chromosomes in tdTomato^+^CD45^−^Ter119^−^ cells in the host is increased, we detected the karyotype of these cells, showing that the number of chromosomes was 40, consistent with wild‐type mice (Figure 5d). These data exclude cell‐cell fusion as the source for HSC‐derived niche cells in host mice.

To further understand the mechanism underlying the regeneration of donor HSCs to niche cells, we examined the distribution of donor fluorescence in the niche cells of the host mice after HSC transplantation. HSC‐regenerated niche cells in the host carried donor tdTomato fluorescence dispersedly displayed in both the cytoplasm and the nucleus, unlike the tdTomato fluorescence pattern with pronounced presence in the cytosol of donor HSCs (Figure 5e). This suggests that transdifferentiation of donor HSCs into niche cells in the host involves reprogramming of the spatial expression of the donor genome.

Taken together, all of the above data (Figure 5) are in agreement with the observation by lineage‐tracing analysis and transcriptomic profiling (Figure 4) that “nonhematopoietic” niche cells are transdifferentiated from young donor HSCs.

### 2.6 Transplantation of HSCs reversed the decline in the bone marrow niche in leukemia patients

**FIGURE 6:**
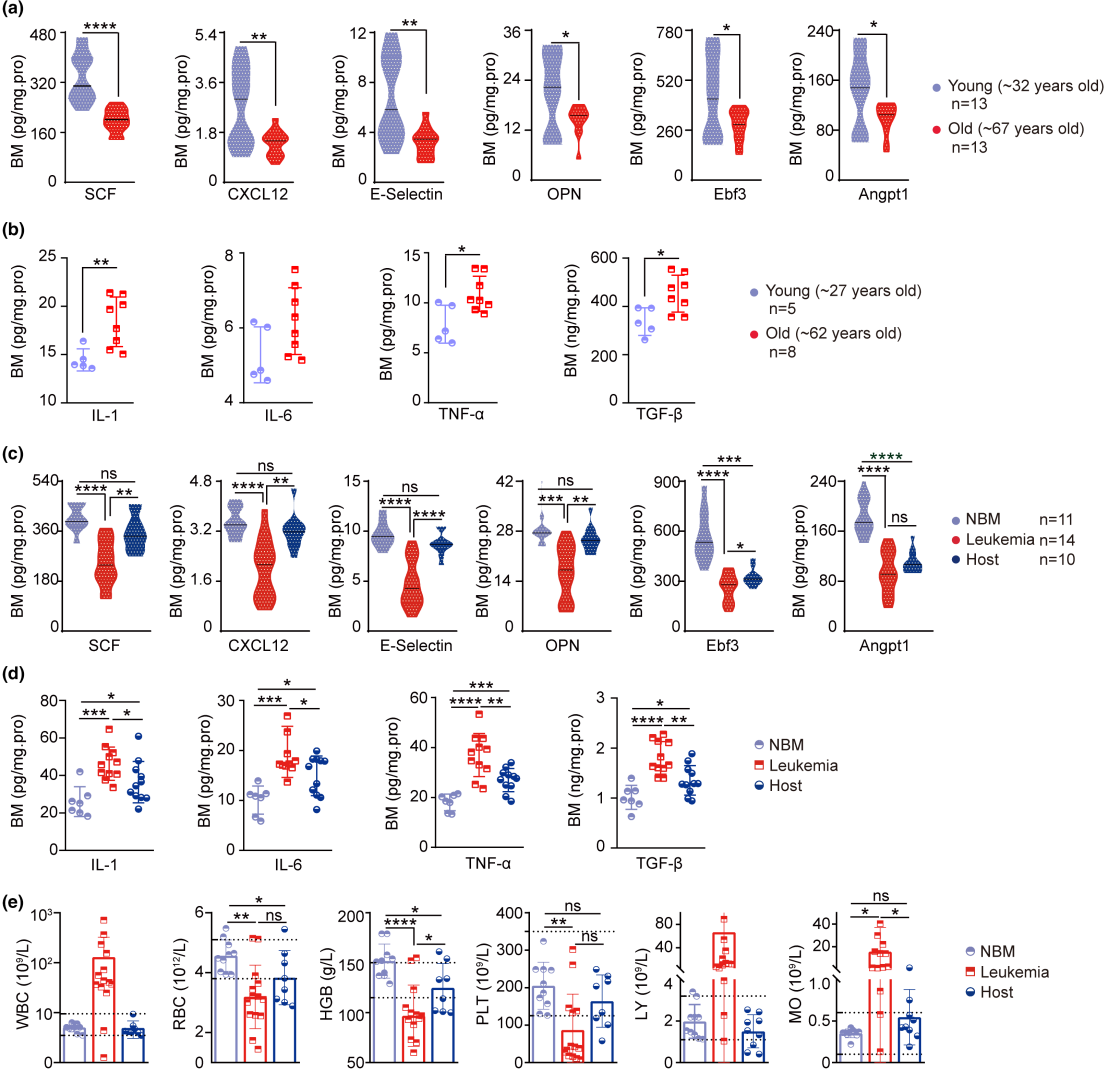
Donor HSCs reversed aged or declined bone marrow niche in leukemia patients. (a) Human bone marrow was collected from the young and old groups. Bone marrow niche factors, including SCF, CXCL12, E‐selectin, OPN, Ebf3, and Angpt1, were quantified by ELISA. (b) Determination of inflammatory factors by ELISA in young and old groups. (c) Determination of niche factors in healthy and leukemia patients before and after HSC transplantation. (d) Determination of inflammatory factors in healthy and leukemia patients before and after HSC transplantation. (e) Determination of peripheral blood counts in healthy individuals and leukemia patients before and after HSC transplantation. HGB, hemoglobin; LY, lymphocytes; MO, monocytes; PLT, platelets; RBC, red blood cells; WBC, white blood cells. ^ns^ *p* > 0.05; *p < 0.05; **p < 0.01; ***p < 0.001; ****p < 0.0001; unpaired two‐tailed *t* test. All error bars indicate SD.

Receiving HSC transplantation from an old donor is associated with poorer outcome in patients diagnosed with hematological malignancies (Bastida et al., 2015; Murthy et al., 2022). This is in accordance with our result that HSCs from aged mice failed to restore niche function and hematopoiesis in the host (Figure 2). However, direct evidence of bone marrow niche aging in humans is inadequate. To address this, we examined healthy young (average age 32) and old (average age 67) humans with their bone marrow (Table S1) and found that the function of the bone marrow niche declined in the old group, with significantly reduced niche factors, including SCF, CXCL12, E‐selectin, OPN, Ebf3, and Angpt1, and significantly increased inflammatory factors, including IL‐1, IL‐6, TNF‐α, and TGF‐β, compared with the young group (Figure 6a,b). The results indicate that the bone marrow niche in older people declines with a reduced capacity to generate niche factors, consistent with observations in mice.

Leukemia patients are characterized by impaired hematopoiesis. As expected, niche factors in various types of leukemia, including acute myeloid leukemia (AML), chronic myeloid leukemia (CML), and acute lymphoblastic leukemia (ALL), were significantly decreased (Figure 6c), indicating that leukemia patients have an aged or declining hematopoietic niche. To test whether donor HSC‐driven renewal or repair of the niche in mice can be recapitulated in humans, we collected bone marrow samples of leukemia patients transplanted with HSCs from healthy young donors. Consistent with the mouse results, HSC transplantation in humans restored the protein expression levels of the above six niche factors (Figure 6c), and inflammatory factors, including IL‐1, IL‐6, TNF‐α, and TGF‐β, in the bone marrow were normalized in HSC‐transplanted leukemia patients (Figure 6d), together indicating the functional restoration of aged or declined niche by young donor HSCs in humans. Along with the restoration of bone marrow niche function, peripheral blood counts were normalized in HSC‐transplanted leukemia patients (Figure 6e).

## 3 DISCUSSION

The bone marrow niche regulates HSC homeostasis through cell–cell interactions and, in particular, the secretion of niche factors. A decrease in niche function promotes HSC aging, myelofibrosis, transformation, and eventually the development and progression of various hematological malignancies (Duarte et al., 2018; Leimkühler et al., 2021; Man et al., 2021; Schepers et al., 2015; Verovskaya et al., 2019). In aged niche, MSCs and ECs fail to express sufficient niche factors to support HSC function (Poulos et al., 2017; Verovskaya et al., 2019; Winkler et al., 2012). Apart from decreased expression of niche factors, niche aging can also be accelerated by increased inflammatory signaling (Kovtonyuk et al., 2016; Mitroulis et al., 2020). In patients or animal models, leukemic cells can directly remodel and inhibit the bone marrow niche, which in turn suppresses HSCs (Arranz et al., 2014; Duan et al., 2014; Duarte et al., 2018; Hameed et al., 2014; Hanoun et al., 2014; Hawkins et al., 2016). Improving the niche by transplantation of ECs (Poulos et al., 2017) or pharmacological revitalization of MSCs or sympathetic cells (Ho et al., 2019; Maryanovich et al., 2018; Nakahara et al., 2019) significantly reverses the aging of old HSCs and promotes HSC expansion and hematopoiesis.

A single transplanted donor HSC is capable of reconstituting the entire hematopoietic system in lethally irradiated mice (Osawa et al., 1996; Uchida et al., 1994), and in zebrafish, HSCs were found to trigger physical remodeling of the perivascular niche to form a surrounding pocket (Tamplin et al., 2015). An important but unanswered question pertinent to clinical significance thus far is what type of cells is responsible for the renewal of the damaged niche in the irradiated host bone marrow and how the host niche is regenerated in the course of HSC engraftment for recovery of hematopoiesis in irradiated mammals. An extended question to the above is whether physiologically or pathologically aged niche can clinically be revitalized in adult bone marrow by donor HSC transplantation. Our recent study showed that HSCs are indispensable in maintaining the macroenvironment in the bone marrow since impairment in HSCs by genetic intervention damages H‐vessels in the bone and promotes osteoporosis, a typical bone aging‐associated syndrome (Yuan et al., 2020).

**FIGURE 7:**
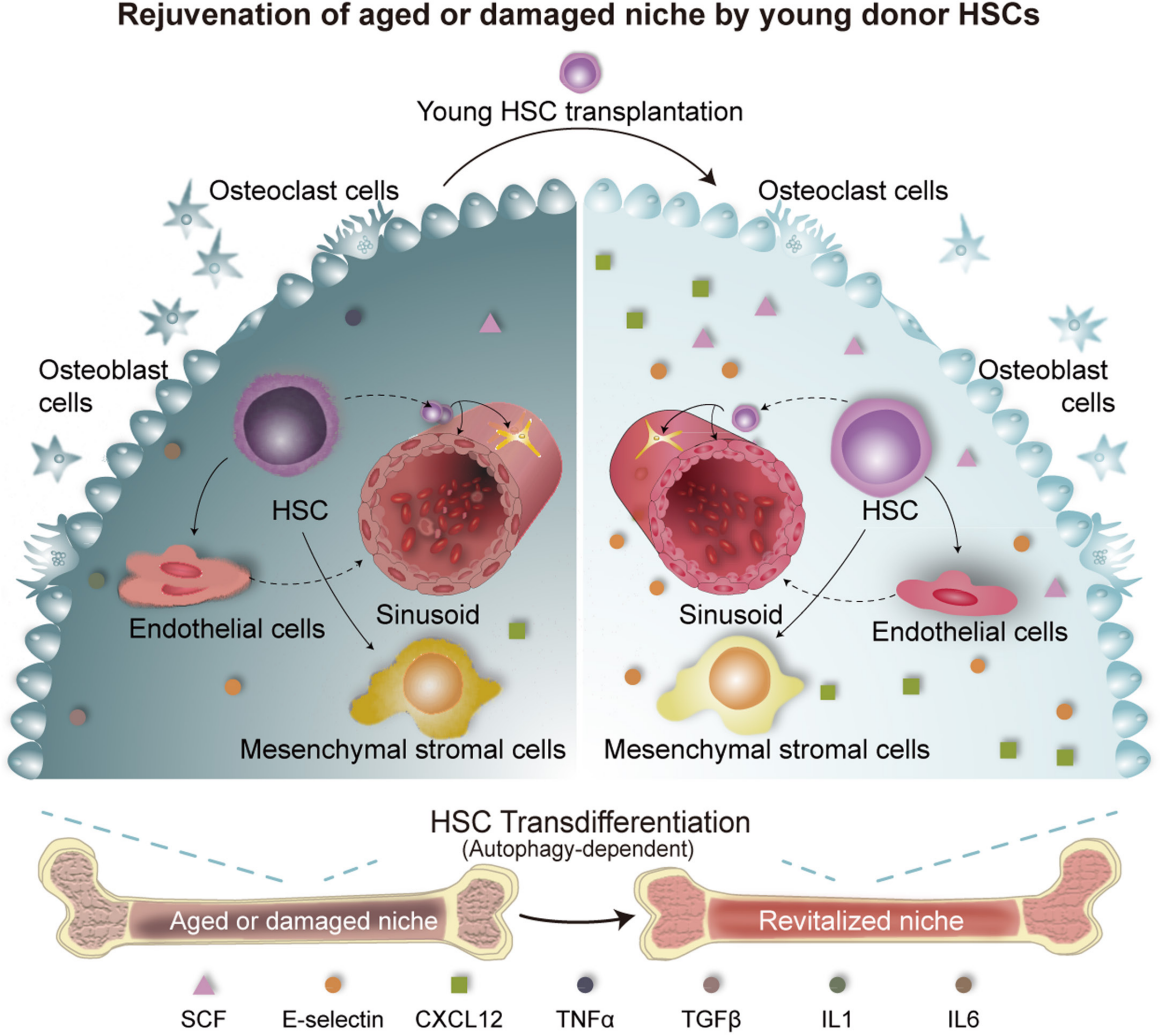
Schematic cartoon illustrating that aged or damaged bone marrow can be rejuvenated or repaired by young donor HSCs. Bone marrow niche aging is characterized by the accumulation of silenced and dysfunctional niche cells. Young donor HSCs are capable of regenerating aged or damaged “nonhematopoietic” niche cells. Transdifferentiation of young donor HSCs to their niche cells in the host depends on autophagy machinery integrity.

In this study, we found that young donor HSCs are capable of reversing a damaged or an aged niche, the microenvironment for HSC activity in the host bone marrow. Using a donor lineage fluorescence‐tracing mouse model in which the progeny cells of fluorescent HSCs can be tracked along with their differentiation trajectory in the host and a niche‐defective model in which bone marrow HSCs and the niche are physiologically aged or functionally impaired by autophagy disruption or by irradiation, we provide evidence that MSCs and ECs, the two major niche cell members in the host, previously regarded as “nonhematopoietic,” originate from donor HSCs (outlined in Figure 7).

Several studies suggest niche lesions as initiating cascades in hematological malignancies (Duhrsen & Hossfeld, 1996; Méndez‐Ferrer et al., 2020). An inflammatory microenvironment with reduced niche factors is an important predisposition factor for many hematological disorders as we age. For example, abnormal alterations in MSCs were observed in patients with myelodysplastic syndrome and acute myeloid leukemia (Blau et al., 2007; Kim, Jekarl, et al., 2015; Kim, Shim, et al., 2015; von der Heide et al., 2017). Dysfunctional niches facilitate mutant hematopoietic cell survival and expansion, leading to malignancy development and progression, and possibly protect malignant cells from chemotherapy, ultimately leading to relapse (Duhrsen & Hossfeld, 1996; Méndez‐Ferrer et al., 2020). Overexpression of transcription factors in MSCs was attempted to improve niche function (Nakahara et al., 2019). Although the young niche in the recipient mice partially restored the transcriptional profile of aged donor HSCs, it was unable to normalize the DNA methylation profiles and function of HSCs (Kuribayashi et al., 2021). In our present study, transplantation of young donor HSCs was shown to improve aged or damaged niche in both animal models and leukemia patients. This is particularly important because patients with hematological malignancies often receive radiotherapy and long‐term chemotherapy, which inevitably harm and even severely impair the bone marrow hematopoietic niche. Therefore, transplantation of younger HSCs may play a dual role in the reconstitution of both hematopoiesis and the hematopoietic niche.

The molecular mechanism by which HSCs are transdifferentiated into their niche cells was not elucidated in this study. However, the possibility of phagocytosis can be precluded because the HSC‐derived niche cells MSCs and ECs in the host did not harbor markers (F4/80^+^CD11b^+^) specific to macrophages, thus excluding false signals for colocalization of the markers from macrophages and HSCs. HSC‐derived cells maintained a homozygous genome and diploidy; confocal microscopy did not find coenocytes; karyotype assay confirmed the normal morphology of the host niche cells and number of chromosomes, thereby ruling out the possibility of cell‐cell fusion to give false signals (Figure 5a,b,d).

Therefore, we argue that transdifferentiation may be responsible for the remodeling of donor HSCs to their niche cells. In addition to donor lineage fluorescence‐tracing analysis, bioinformatics analysis revealed transcriptomic imprinting from donor HSCs in host niche cells. The transdifferentiation trajectory may be triggered by aging or damaging stresses, particularly by the accumulation of inflammation in the niche, which is in agreement with the pioneering report on bone marrow‐derived cell transdifferentiation to nonbone marrow cells by Krause and colleagues (Harris et al., 2004; Krause et al., 2001). Bone marrow consists of a long array of cell types with and without blood lineage. It is not surprising that certain types of bone marrow cells can fuse with cells from nonhematopoietic organs (Alvarez‐Dolado et al., 2003; Terada et al., 2002). Our results suggest that donor HSCs are responsible for the transdifferentiation potential of bone marrow cells into niche cells. Recently, donor HSCs were found to differentiate into 28 cell types in myeloablated recipients at an early stage one week after transplantation (Dong et al., 2020), a number much more than that currently known as “hematopoietic cells,” suggesting that HSC programming for hematopoietic lineage differentiation and HSC transdifferentiation for regeneration of bone marrow niche cells may start at an early stage after HSC transplantation.

Transdifferentiation of donor HSCs into niche cells in the host may involve reprogramming of the spatial expression of the donor genome since the tdTomato fluorescence pattern varies between donor HSCs and HSC‐derived niche cells (Figure 5c,e). Recent studies have found mitochondrial transfer from Cx43‐expressing hematopoietic progenitors to the stroma (Golan et al., 2020), and microvesicles transfer mitochondria to endothelial cells and brain slice neurons (D'Souza et al., 2021). Our results show that niche cells in the host carry chromosomal genes and transcriptomic imprinting from young donor HSCs (Figure 4). Since no nuclear transfer of HSCs has been reported via exosome transfer, whether exosomal transfer is part of the mechanisms responsible for transdifferentiation remains an open question.

Autophagy has been documented to decelerate hematopoietic aging (Fang et al., 2020; Ho et al., 2017). The present study shows that regeneration of the niche by HSC transdifferentiation depends on the integrity of the autophagy machinery since deletion of *Atg7*, an essential autophagy gene, disabled the capacity of HSCs to generate niche cells in the host; in particular, autophagy defects in the hematopoietic system apparently cause bone marrow niche aging (Figure 1). Therefore, the maintenance of the niche by HSCs depends on autophagy in both transplant and nontransplant settings.

In summary, the present study establishes that bone marrow aging is characterized by extensively silenced and dysfunctional niche cells and that young donor HSCs can serve as the parental source for regenerating pathologically damaged or physiologically aged niches, supporting an increased multipotency of HSCs for transdifferentiation to the “nonhematopoietic” lineage in the case of niche damage or decline. Young donor HSCs can flexibly orchestrate the balanced production of their progeny cells between the blood lineage and bone marrow niche lineage, thereby securing niche‐supported hematopoiesis. Therefore, young HSC transplantation may be used to improve both HSC hematopoiesis and its supporting niche in leukemia patients. Nevertheless, future study is warranted to determine the molecular mechanism driving such a transdifferentiation trajectory.

## 4 MATERIALS AND METHODS

### 4.1 Mouse models and xenografts

C57BL/6J, CD45.1, *Rosa* ^mT/mG^ and *Atg7* ^f/f^;Vav‐iCre mice were used in this study. *Rosa* ^mT/mG^ mice were generated by the laboratory of Dr. Liqun Luo (Muzumdar et al., 2007). Mice with *Atg7* gene deficiency in hematopoiesis were obtained by crossing *Atg7* ^f/f^ mice kindly from Dr. Komatsu, Japan (Komatsu et al., 2005) with *Vav‐iCre* mice (Jackson Laboratory). For genotypic analysis, DNA was extracted by a Genomic DNA Mini Preparation Kit with a Spin Column. For transplantation assays, 2000 HSCs with 200,000 bone marrow cells were transplanted into each recipient with irradiation (9 or 5 Gy). The recipients were killed 16 weeks after transplantation, and multilineage reconstitution was monitored in the bone marrow. The mice were bred and housed in the specific‐pathogen‐free animal facilities of Soochow University. All experiments with animals were in compliance with the institutional protocols for animal welfare and approved by the Ethics Committee of Soochow University.

### 4.2 Human samples

Human samples were collected from the affiliated hospitals of Soochow University in accordance with the University's code on Medical Ethics. The sample information is detailed in Table S1.

### 4.3 DNA isolation and genotyping

Genomic DNA of the indicated cells was isolated using a Genomic DNA Mini Preparation Kit with a Spin Column (Beyotime). Genotyping via PCR was performed using the following primers:

*Atg7*‐F:5′‐CATCTTGTAGCACCTGCTGACCTGG‐3′,*Atg7*‐R:5′‐CCACTGGCCCATCAGTGAGCATG‐3′, *Loxp*‐R:5′‐GCGGATCCTCGTATAATGTATGCTATACGAAGTTAT‐3′; *MTG*‐F:5′‐CTCTGCTGCCTCCTGGCTTCT‐3′, *MTG*‐R1:5′‐CGAGGCGGATCACAAGCAATA‐3′, *MTG*‐R2:5′‐TCAATGGGCGGGGGTCGTT‐3′; *Vav‐icre‐*F:5′‐AGATGCCAGGACATCAGGAACCTG‐3′, *Vav‐icre‐*R:5′‐ATCAGCCACACCAGACACAGAGATC‐3′; *Ddx3y*‐F:5′‐CCAATAGCAGCCGAAGTAGTGGTAG‐3′, *Ddx3y*‐R:5′‐TTAGGGTACAACCAAGCAGGAAGTG‐3′; *β‐actin‐*F:5′‐TCGTGCGTGACATCAAAGAGA‐3′, *β‐actin‐*R:5′‐GAACCGCTCGTTGCCAATA‐3′.

### 4.4 Bone immunostaining and image flow cytometry

Hind limbs were collected from the indicated mice, and the soft tissue was cleaned. Femurs and tibias were fixed in 4% paraformaldehyde (PFA) overnight at 4°C and then decalcified in EDTA (5 M, pH 8.0) for 2 weeks. For dehydration, the bones were incubated in 2% polyvinylpyrrolidone (PVP) and 20% sucrose in PBS for 1 week at 4°C. Finally, tissues were embedded in O.C.T. T tissue freezing medium (Leica) and cut into 20 μm‐thick sections for hematoxylin and eosin (H&E) staining, toluidine blue staining, and immunohistochemistry. Thick sections were rinsed with PBS and postblocked with 5% donkey serum (DS; Sigma) in 0.1% Triton X‐100/PBS for 2 h at room temperature. For endothelial cell staining, anti‐CD31 antibody (Cat: ab28364; Abcam) was used at a 1:800 dilution in 5% DS 0.1% Triton X‐100/PBS overnight at 4°C. For adipocyte staining, anti‐Perilipin A/B antibody (Cat: P1873; Sigma) was used at a 1:800 dilution in 2% DS 0.1% Triton X‐100/PBS overnight at 4°C. For osteoblast staining, anti‐osteopontin/OPN antibody (Cat: AF808; RD) was used at a 1:800 dilution in 2% DS 0.1% Triton X‐100/PBS overnight at 4°C. After 3 washes with 0.1% Triton X‐100/PBS, the sections were incubated with Alexa Fluor 488‐conjugated goat anti‐rat IgG (Cat: ab150077, Abcam) secondary antibody and 0.1% DAPI (4′,6‐diamino‐2‐phenylindole; Sigma). After washing 3 times, the slides were covered. Images were taken on an Olympus Confocal FV3000. For image flow cytometry, endothelial cells and mesenchymal stem cells were stained with their markers (endothelial cells: CD45^−^Ter119^−^CD31^+^, mesenchymal stem cells:CD45^−^Ter119^−^CD31^−^PDGFRα^+^), respectively, and conjugated with FITC. The colocalization of tdTomato fluorescence and these stromal cells was quantified by Image Flow Wizard software.

### 4.5 Micro‐CT analysis

The hind limb from the same side of each mouse was fixed in 2% polyvinylpyrrolidone (PVP) for 48 h, and the limbs were scanned on a SkyScan micro‐CT system (SkySacn, Antwerp, Belgium).

### 4.6 Flow cytometry analysis and cell sorting

BM cells from *Atg7* ^f/f^;Vav‐iCre mice, control littermates, and old mice were collected. The HSCs were analyzed after exclusion of lineage (Lin)‐positive cells using a Lineage Cell Depletion Kit (MiltenyiBiotec) and after labeling with Sca‐1, c‐kit, CD150 and CD48 antibodies. For stromal cell analysis, bone marrow cells were labeled with antibodies against CD45 and Ter119 (BD Biosciences). For mesenchymal cell analysis, bone marrow cells were labeled with antibodies against CD45, Ter119, CD31, and PDGFRα (BD Biosciences). For endothelial cell analysis, bone marrow cells were labeled with antibodies against CD45, Ter119, and CD31. After incubation, the labeled cells were analyzed on a FACSCalibur flow cytometer (BD). For cell purification, CD45^−^Ter119^−^CD31^−^PDGFRα^+^ mesenchymal cells and CD45^−^Ter119^−^CD31^+^ endothelial cells were sorted on a cell sorter (FACSAria; BD).

### 4.7 RNA isolation and RT–PCR assay

Total RNA was extracted from hematopoietic cells, nonhematopoietic stromal cells, endothelial cells, and mesenchymal cells using a MicroElute Total RNA Kit (OMEGA). One microgram of total mRNA was reverse transcribed into complementary DNA (cDNA) using a RevertAid First Strand cDNA Synthesis Kit (Thermo Fisher Scientific). Quantitative real‐time (RT) polymerase chain reaction (PCR) was performed using LightCycler 480 SYBR GreenI Master Mix (Roche, 04707516001). Data were collected and analyzed on a LightCycler480II (Roche). The primers used included the following:

*SCF*‐F: 5′‐CCCTGAAGACTCGGGCCTA‐3′;

*SCF*‐R: 5′‐CAATTACAAGCGAAATGAGAGCC‐3′;

*CXCL*12‐F: 5′‐GCTCTGCATCAGTGACGGTA‐3′;

*CXCL*12‐R: 5′‐TAATTACGGGTCAATGCACA‐3′;

*E‐selectin‐*F: 5′‐TGAACTGAAGGGATCAAGAAGACT‐3′;

*E‐selectin‐*R: 5′‐GCCGAGGGACATCATCACAT‐3′;

*Ebf3‐*F: 5′‐CGAAAGGACCGCTTTTGTGC‐3′;

*Ebf3‐*R: 5′‐AGTGAATGCCGTTGTTGGTTT‐3′;

*OPN‐*F: 5′‐ACCCTGGCTGCGCTCTGTCTCT‐3′;

*OPN‐*R: 5′‐GATGCGTTTGTAGGCGGTCTTCA‐3′;

*Angpt1*‐F: 5′‐CAT TCT TCG CTG CCA TTC TG‐3′;

*Angpt1*‐R: 5′‐GCA CAT TGC CCA TGT TGA ATC‐3′;

*CD*105‐F: 5′‐TTGAATGGCAACCACGAGC‐3′;

*CD*105‐R: 5′‐GAGCCTGACGGGAAACTGAT‐3′;

*CD*73‐F: 5′‐GGCTGCTTCTCGCACTGA‐3′;

*CD*73‐R: 5′‐CTGGTACTGGTCTCCGGC‐3′;

*CD*29‐F: 5′‐TGGTCAGCAACGCATATCT‐3′;

*CD*29‐R: 5′‐TTGTCCATCATTGGGTAAAAC‐3′;

*CD*44‐F: 5′‐CGTCCAACACCTCCCACTAT‐3′;

*CD*44‐R: 5′‐AGCCGCTGCTGACATCGT‐3′;

*CD90*‐F: 5′‐GGTGAACCAAAACCTTCGCC‐3′;

*CD90*‐R: 5′‐ACACTTGACCAGCTTGTCTCT‐3′.

### 4.8 RNA sequencing analysis

MSCs (CD45^−^Ter119^−^CD31^−^PDGFRα^+^) were sorted from 10‐week‐old mice, 72‐week‐old mice and *Atg7* ^−/−^ mice. HSCs were sorted from 8‐week‐old *Rosa* ^mT/mG^ mice, tdTomato^+^ and tdTomato^−^stromal cells were sorted from host mice, and total stromal cells were sorted from WT mice. The experimental procedure was as follows: (1) mRNA enrichment and purification: Oligo dT selection to enrich the mRNA (for total RNA extracted from human whole blood, globin mRNA is depleted); (2) RNA fragmentation and cDNA synthesis (second‐strand cDNA synthesis with dUTP instead of dTTP); (3) end repair, addition of A and adaptor ligation; (4) PCR; (5) circularization and DNB; and (6) sequencing on the DNBSEQ platform. Sequencing data filtering used the software SOAPnuke developed by BGI independently for filtering, and these data were subjected to quality control (QC) to guarantee suitability for analysis. Unsupervised hierarchical clustering on expression profile data used in the clustering process was filtered with TPM > 20 (in at least one sample), and we selected the 2000 most variable genes to conduct cluster analysis. Complete‐linkage hierarchical clustering was used to cluster the expression profiles, and the distance metric we used was Euclidean distance. The expression heatmap was plotted using filtered TPM expression and was normalized by gene. The clustering result was visualized as dendrograms in the expression heatmap. Similar gene expression patterns were clustered into 5 clusters using *k*‐Means. Enrichment analysis was conducted to investigate the function of each clustered module.

### 4.9 Statistical analysis

Statistical analyses were performed using SPSS version 25.0. The clinical data of leukemia patients and healthy individuals were collected with blood cell count, classification, sex, and age. Experimental data are presented as the means ± standard deviations (SDs), which were evaluated using unpaired Student's *t* tests. *p* values are reported as nonsignificant when *p* ≥ 0.05. All statistical analyses were performed using GraphPad Prism v. 8 software. Graphs were plotted using Adobe Illustrator CS.

## AUTHOR CONTRIBUTIONS

Na Yuan and Jianrong Wang designed the experiments and analyzed the data. Na Yuan, Li Ji, and Wen Wei performed most of the experiments. Jiawei Qian performed bioinformatics analysis. Wen Wei, Li Xu, Lei Li, Chen Zhao, Xueqin Gao, and Zhicong Jin performed genotyping analysis and transplantation experiments. Hong Liu, Yulong He, Longhai Tang, and Mingyuan Wang provided human samples or reagents. Jianrong Wang conceived and supervised the study. Na Yuan, Yixuan Fang, and Jianrong Wang wrote the manuscript. All authors discussed the results and commented on the manuscript.

## CONFLICT OF INTEREST STATEMENT

None declared.

## Supporting information


Data S1.

## Data Availability

All sequencing data have been uploaded to the Gene Expression Omnibus database. The accession numbers are pending approval.
