# Supplementary material for: Young donor hematopoietic stem cells revitalize aged or damaged bone marrow niche by transdifferentiating into functional niche cells
Source: Aging Cell. 2023 May 24;22(8):e13889. doi: 10.1111/acel.13889 (PMC10410009; doi:10.1111/acel.13889)
Supplement: Supplementary file 1 — Data S1. [file ACEL-22-e13889-s001.pdf]

Supplemental material

Young donor hematopoietic stem cells revitalize aged or damaged bone marrow niche by transdifferentiating into functional niche cells

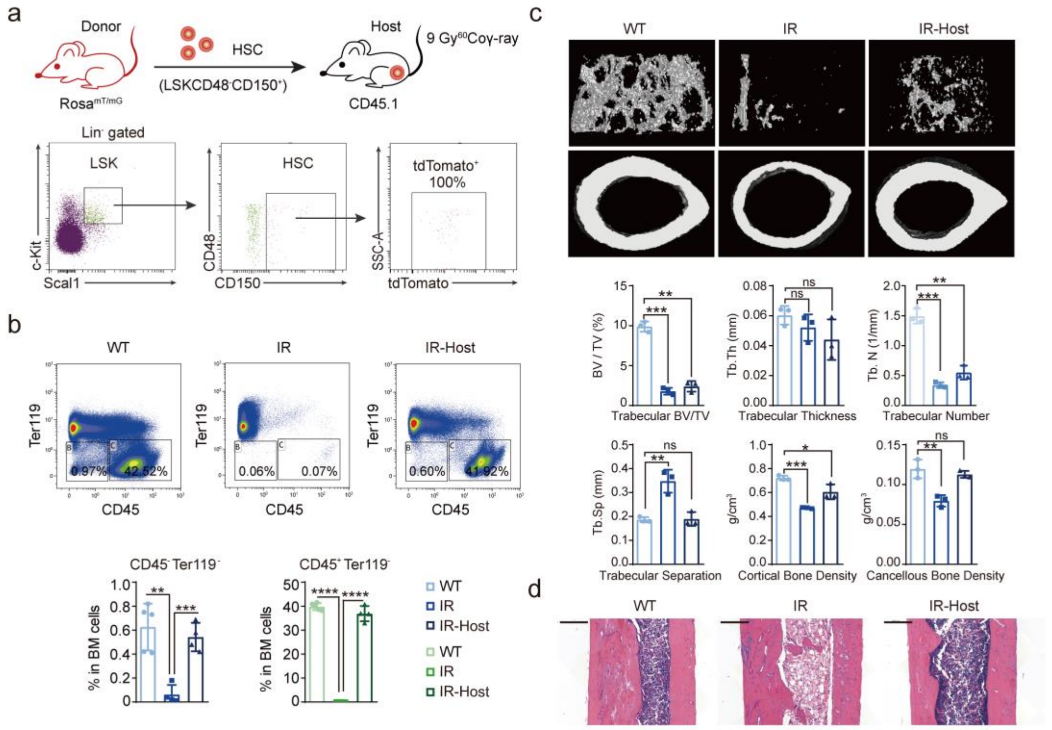

Figure S1. HSCs rebuilt the bone marrow "nonhematopoietic" niche and reconstituted hematopoiesis in the host of endogenous bone marrow niche previously disrupted by irradiation. **a**, Schematic procedure of the transplantation. Nine Gy-irradiated C57 mice were transplanted with 2000 HSCs from *Rosa*$^{mT/mG}$ mice and 200,000 whole bone marrow cells from CD45.1 mice. The host bone marrow cells were analyzed at 12 weeks posttransplantation. **b**, Representative flow cytometry plots (top) with quantification of CD45^{+}Ter119^{-} cells and CD45^{-}Ter119^{-} cells in the bone marrow of WT, IR(irradiation) and IR-Host mice (bottom). **c**, Representative micro-CT reconstructed three-dimensional pictures of femur trabecular (top) and cortical bone (bottom). Femora were collected from 10-week-old WT, IR(irradiation) and IR-Host mice. Measurement of distal femur spatial structure parameters included trabecular relative bone volume (BV/TV), trabecular thickness (Tb. Th), trabecular number (Tb. N), trabecular separation (Tb. Sp), cortical bone density (g/cm^3) and cancellous bone density (g/cm^3) from WT, IR and IR-Host mice (lower). **d**, H&E staining of femur paraffin sections. Femurs were collected from 10-week-old WT, IR and IR-Host mice for paraffin sectioning and immunohistochemistry. Bar=200 μm. n=3, from three independent experiments. ns: $P > 0.05$; *: $P < 0.05$; **: $P < 0.01$; ***: $P < 0.001$; ****: $P < 0.0001$; unpaired two-tailed t test. All error bars indicate SD.

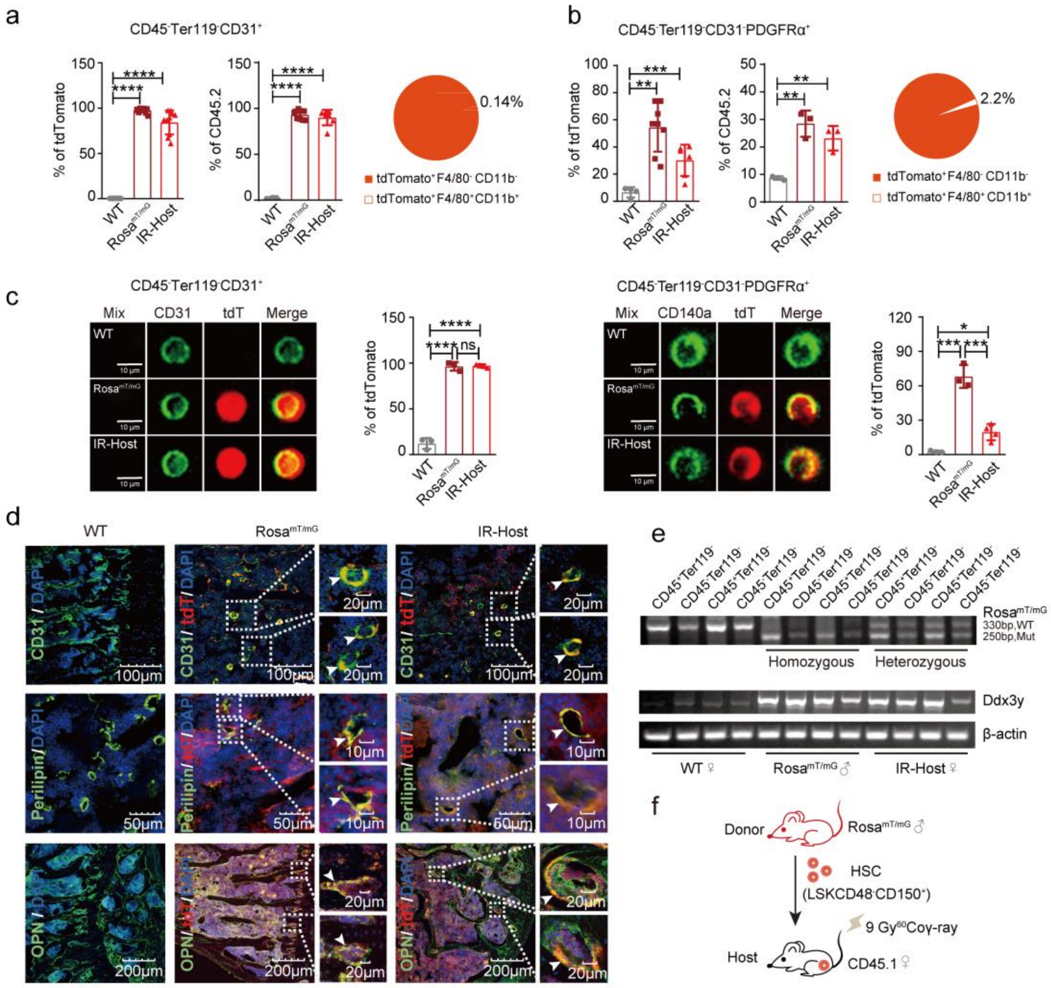

Figure S2. Host bone marrow "nonhematopoietic" niche cells, previously damaged by irradiation, carried the markers of donor HSCs. a-b, The percentages of bone marrow tdTomato^{+} cells and the expression of CD45.2 in CD45^{-}Ter119^{-}CD31^{+} and CD45^{-}Ter119^{-}CD31^{-}PDGFRα^{+} cells from the indicated mice 16 weeks after transplantation (histogram). The percentages of bone marrow macrophages in the indicated tdTomato^{+}CD45^{-}Ter119^{-}CD31^{+} and tdTomato^{+}CD45^{-}Ter119^{-}CD31^{-}PDGFRα^{+} cells (pie chart). **c**, Imaging flow analysis of CD45^{-}Ter119^{-}CD31^{+} and CD45^{-}Ter119^{-}CD31^{-}PDGFRα^{+} cells from WT, *Rosa*$^{mT/mG}$, and IR-host mice (left). CD45^{-}Ter119^{-}CD31^{+} and CD45^{-}Ter119^{-}CD31^{-}PDGFRα^{+} cells (green dots), *Rosa*$^{mT/mG}$ fluorescence (red dots), merge (yellow dots). Quantification of merged cells in the indicated mice 16 weeks after transplantation (right). **d**, Representative confocal images of bones from WT, *Rosa*$^{mT/mG}$, and IR-host mice stained for CD31^{+} vasculature endothelial cells, Perilipin^{+} adipocytes, and osteoblasts with anti-OPN and 4′,6- diamidino-2-phenylindole (DAPI). High magnifications (right) show the enlarged colocation regions. Arrows mark the merged cells. n=3, from three independent experiments. **e**, PCR analysis of the presence of the *Ddx3y* gene in CD45^{-}Ter119^{-} cells and CD45^{+}Ter119^{-} cells in *Atg7*$^{-/-}$ female, *Rosa*$^{mT/mG}$ male, and IR-Host female mice. *Atg7*$^{-/-}$ female mice served as a negative control. *Rosa*$^{mT/mG}$ male mice served as a positive control. **f**, Schematic procedure of HSC transplantation. Nine Gy-irradiated CD45.1 mice were transplanted with 2000 HSCs from *Rosa*$^{mT/mG}$ mice and 200,000 whole bone marrow cells from CD45.1 mice. The host bone marrow cells were analyzed at 16 weeks post-transplantation. ns: $P > 0.05$; *: $P < 0.05$; **: $P < 0.01$; ***: $P < 0.001$;

****: $P < 0.0001$; unpaired two-tailed t test. All error bars indicate SD.

Sample	Classification	Sex	Age	Sample	Classification	Sex	Age	Sample	Classification	Sex	Age
1	NBM	Male	24	1	Old	Female	63	1	AML	Female	39
2	NBM	Female	43	2	Old	Female	71	2	AML	Male	39
3	NBM	Male	15	3	Old	Female	91	3	ALL	Male	58
4	NBM	Male	45	4	Old	Female	62	4	AML	Female	39
5	NBM	Male	29	5	Old	Female	55	5	ALL	Female	62
6	NBM	/	/	6	Old	Male	56	6	AML	Male	40
7	NBM	/	/	7	Old	Male	55	7	AML	Male	75
8	NBM	Female	44	8	Old	Female	62	8	CML	Male	63
9	NBM	Female	37	9	Old	Male	56	9	CML	Male	26
10	NBM	Male	32	10	Old	Female	76	10	CML	Male	38
11	NBM	Female	23	11	Old	Female	68	11	CML	Male	47
12	NBM	Male	40	12	Old	Male	78	12	AML	Female	25
13	NBM	Male	45	13	Old	Male	83	13	CLL	Male	31
14	NBM	Female	48	14	Old	Male	52	14	AML	Male	37
15	NBM	Male	32	15	Old	Male	53	1	HSCT-AML	Male	26
16	NBM	Male	49	16	Old	Male	55	2	HSCT-AML	Male	29
17	NBM	Male	40	17	Old	Male	60	3	HSCT-AML	Female	53
18	NBM	Male	45	18	Old	Male	61	4	HSCT-ALL	Female	37
19	NBM	Female	23	19	Old	Female	70	5	HSCT-AML	Male	55
20	NBM	Male	53	20	Old	Female	66	6	HSCT-AML	Male	18
21	NBM	Male	57	21	Old	Female	81	7	HSCT-AML	Male	43
22	NBM	Male	51	25	NBM	Male	28	8	HSCT-AML	Female	21
23	NBM	Female	23	26	NBM	Male	29	9	HSCT-ALL	Male	24
24	NBM	Male	24	27	NBM	Male	31	10	HSCT-AML	Female	50

Table S1. Information on primary samples. Human bone marrow aspirates were obtained from the First Hospital Affiliated to Suzhou Medical College of Soochow University. NBM: normal bone marrow, AML: acute myeloid leukemia, CML: chronic myeloid leukemia, ALL: acute lymphoblastic leukemia, M: male, F: female, BMT: bone marrow transplantation.
